# The importance of microtubule-dependent tension in accurate chromosome segregation

**DOI:** 10.3389/fcell.2023.1096333

**Published:** 2023-01-23

**Authors:** Angela R. Bunning, Mohan L. Gupta Jr.

**Affiliations:** Department of Genetics, Development, and Cell Biology, Iowa State University, Ames, IA, United States

**Keywords:** spindle checkpoint, tension, chromosome segregation, microtubule, kinetochore

## Abstract

Accurate chromosome segregation is vital for cell and organismal viability. The mitotic spindle, a bipolar macromolecular machine composed largely of dynamic microtubules, is responsible for chromosome segregation during each cell replication cycle. Prior to anaphase, a bipolar metaphase spindle must be formed in which each pair of chromatids is attached to microtubules from opposite spindle poles. In this bipolar configuration pulling forces from the dynamic microtubules can generate tension across the sister kinetochores. The tension status acts as a signal that can destabilize aberrant kinetochore-microtubule attachments and reinforces correct, bipolar connections. Historically it has been challenging to isolate the specific role of tension in mitotic processes due to the interdependency of attachment and tension status at kinetochores. Recent technical and experimental advances have revealed new insights into how tension functions during mitosis. Here we summarize the evidence that tension serves as a biophysical signal that unifies multiple aspects of kinetochore and centromere function to ensure accurate chromosome segregation.

## Introduction

**FIGURE 1:**
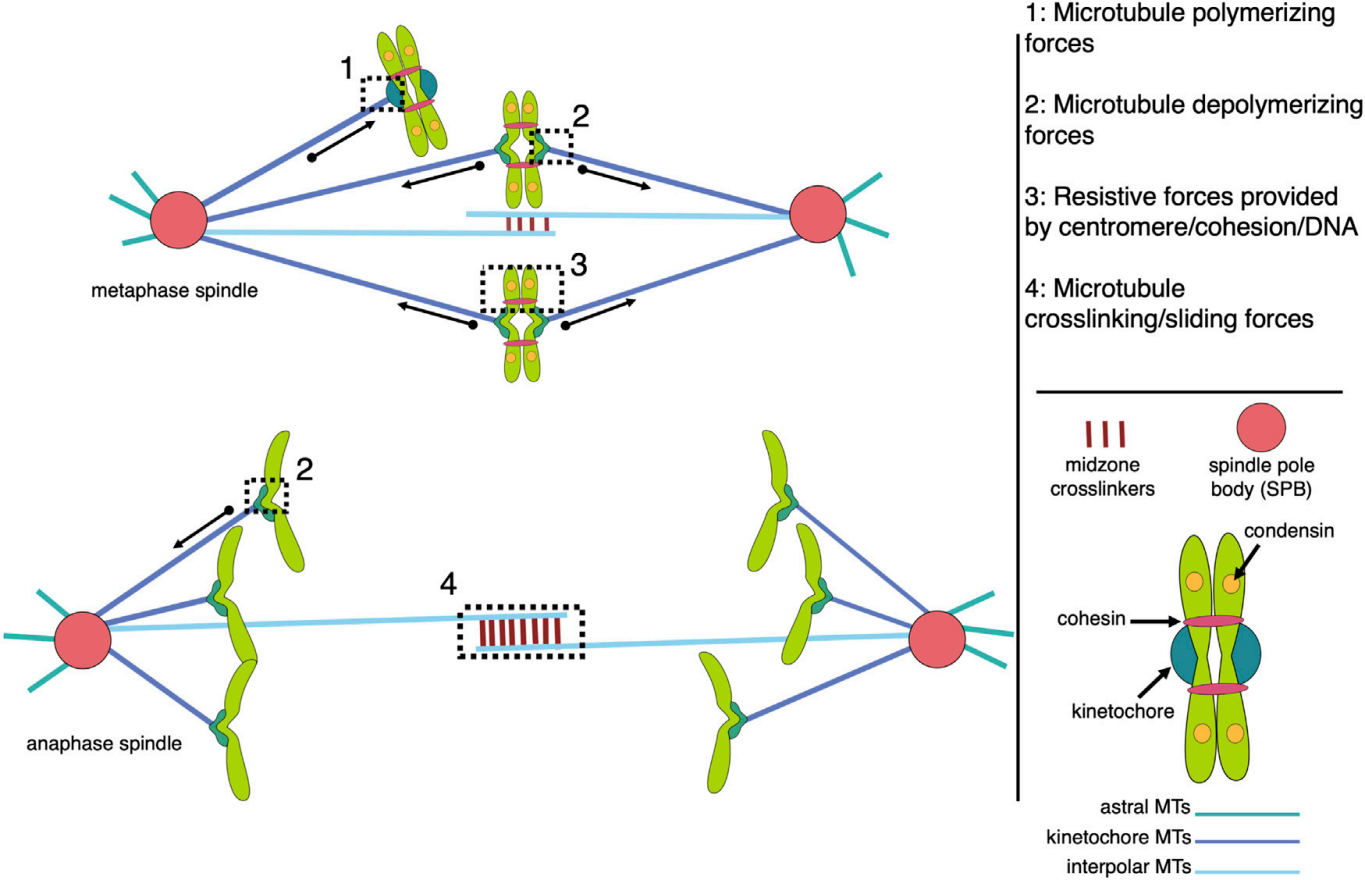
Models of microtubule-associated forces in metaphase and anaphase mitotic spindles. The spindle is composed of three major classes of microtubules (interpolar, kinetochore, and astral), each with unique functions that contribute to the forces generated within the bipolar structure. The forces of note include pushing and pulling forces resulting from microtubule polymerization and depolymerization, respectively, which are largely responsible for chromosome movement (1 and 2), and resistive forces, or tension, generated across pairs of sister kinetochores and centromeres, which are coupled by centromere-associated condensin and cohesin protein complexes (3). MAPs (microtubule associated proteins) crosslink antiparallel interpolar microtubules to create a stable midzone that allows kinesin motor proteins to generate sliding forces that push the spindle poles apart (4). While all four types of forces are active in metaphase spindles, tension across sisters is terminated by cohesion cleavage at the metaphase-to-anaphase transition while anaphase chromosome movement is dominated by microtubule-generated pulling forces.

The mitotic spindle, a highly organized yet morphologically dynamic macromolecular machine composed largely of microtubules and associated proteins, is essential to successfully segregate chromosomes during each round of mitosis. The metaphase spindle has a conserved steady-state structure, which is inherently stable in a bipolar configuration that focuses the microtubules into two poles, crosslinks interpolar microtubules to maintain pole separation, and attaches sister chromatids to kinetochore microtubules from opposite poles (Figure 1).

Microtubules are inherently dynamic polymers composed of tubulin protein, a heterodimer of alpha- and beta-tubulin subunits. These dynamic microtubules are organized and coordinated by the actions of many conserved microtubule associated proteins (MAPs). A key aspect of spindle function is that chromatid pairs will be segregated to opposite poles, and thus into different daughter cells, *via* depolymerizing microtubules, or kinetochore-fibers, by attaching the kinetochores of sister chromatids to microtubules emanating from opposite poles. The kinetochore is a proteinaceous complex that forms on the single centromere of each chromosome. It serves as a physical linkage between the chromosomal centromere and the attached microtubule. When a dynamic microtubule becomes attached to, or captured, by a kinetochore, it can generate a pulling force that creates tension across the sister kinetochores if they are attached to opposite spindle poles. Both the microtubule attachment status and the tension across bipolar attached sister kinetochores serve to ensure chromosomes are accurately segregated during anaphase.

Several decades of work have helped elucidate what proteins mediate the kinetochore-microtubule attachment, how unattached kinetochores act as a signal to delay anaphase onset, and how the phosphorylation of kinetochore proteins regulates the strength of the kinetochore-microtubule attachment. Efforts to discern the mechanisms that sense and respond to microtubule-generated tension at kinetochores have been comparably more difficult due to the interdependency of tension and attachment. Here we provide a brief overview of our understanding of the forces in the bipolar mitotic spindle and how those forces allow dynamic microtubule-kinetochore attachments to generate tension across sister chromatids. We summarize how tension acts as a unifying force that alters kinetochore and centromere structure, mediates Aurora B activity, corrects erroneous attachments, and regulates mitotic progression.

## Forces in the mitotic spindle and associated kinetochore-microtubule tension

The forces acting on and within the mitotic spindle must be balanced to facilitate a stable metaphase configuration with bipolar chromatid attachments (Figure 1). Forces in the spindle can be passive, such as friction or structural elasticity, or active, which requires energy input and can result in mechanical output, such as rearrangements or movement (For a review of all spindle forces, see Nazockdast and Redemann (2020); Dumont and Mitchison (2009). In addition to motor proteins, e.g., kinesins, microtubule polymerization (elongation) and depolymerization (shortening) in the spindle can generate these active forces. Microtubule polymerization and depolymerization are both thermodynamically favorable reactions, relying ultimately on the energy of GTP binding to free tubulin and subsequent hydrolysis within the microtubule polymer (Mitchison and Kirschner, 1984).

The forces generated by a single polymerizing microtubule have been measured to be up to 3–4 pN (Dogterom and Yurke, 1997); (Janson et al., 2003). This force is limited in longer microtubules due to an increase in propensity for buckling (Dogterom and Yurke, 1997), yet microtubule bundling by MAPs can increase overall rigidity while additively increasing their combined force-generating potential (Laan et al., 2008). The so-called polar ejection forces are a well-characterized example of microtubule polymerization-driven forces in the mitotic spindle. These forces, which push chromosomes from near the poles toward the central region of the spindle to aid in chromosome congression, are generated by a combination of polymerizing microtubules and kinesins interacting with chromosome arms (Brouhard and Hunt, 2005); (Ke et al., 2009). In *Drosophila*, polar ejection forces are generated by microtubule polymerization, while NOD, a kinesin-10 motor, couples growing microtubule tips with chromosome arms supporting a polymer ratchet mechanism (Cochran et al., 2009). NOD also has its own plus-end directed motility and plus-end tracking ability *via* EB1 interaction (Ye et al., 2018). Thus, NOD has two force generating activities that contribute to polar ejection forces (Ye et al., 2018). In HeLa cells, the kinesin-10, Kid, links polymerizing microtubule tips with chromosome arms, while a second motor, the kinesin-4 Kif4A, regulates microtubule growth (Stumpff et al., 2012). Another function of microtubule polymerization-derived force is to position microtubule organizing centers within the cell. For example, microtubules pushing against opposite sides of the cell cortex work to center the nucleus in fission yeast (Tran et al., 2001). More recently, microtubules pushing against the cell cortex were shown to maintain metaphase spindle positioning at the cell center in *C. elegans* embryos (Garzon-Coral et al., 2016).

**FIGURE 2:**
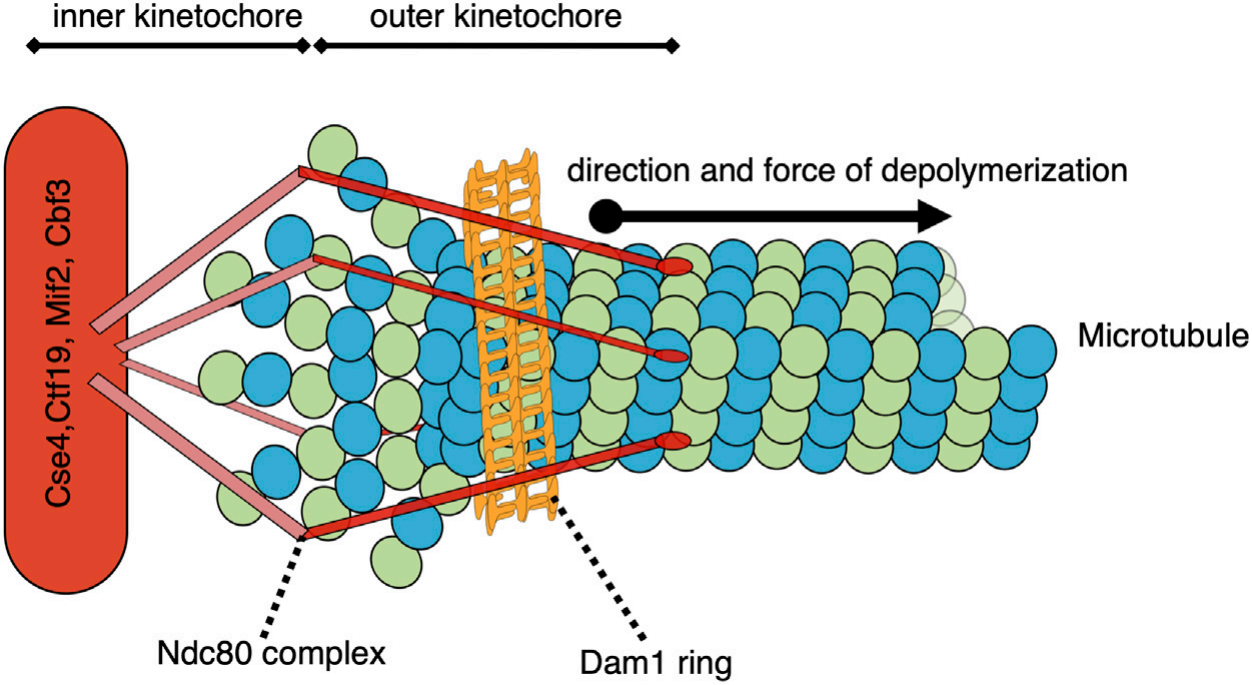
Model of a simplified yeast kinetochore-microtubule attachment with a catch bond-like connection. While many proteins comprise the kinetochore and/or participate in the microtubule-kinetochore attachment, two structures of note are the Ndc80 complex and the Dam1/DASH complex ring. As the end-on microtubule depolymerizes into tubulin heterodimers, the 13 protofilaments each curve outward. These protofilaments are constrained within the collar formed by the ring of Dam1/DASH complexes, and their bending drives the collar further onto the depolymerizing microtubule. The Dam1/DASH ring then pulls the associated centromere/kinetochore *via* the Ndc80-mediated coupling. This pulling force could potentially be sensed by the Dam1/DASH complex, Ndc80, other outer or inner kinetochore proteins, centromere-associated proteins, DNA, or proteins involved in the coupling between the sister centromeres.

The forces associated with microtubule depolymerization are significantly larger than those resulting from polymer growth. In a pioneering study, the wave of curling protofilaments that accompanies depolymerizing microtubules tips was measured to produce 0.5 pN on a bead positioned on one side of the depolymerizing polymer (Grishchuk et al., 2005). In an updated adaptation of this “wave assay”, these forces were measured to be between 8–16 pN (Driver et al., 2017). The total pulling force generated by all protofilaments of a depolymerizing microtubule remains to be directly measured, but has been extrapolated to be 30–65 pN (Grishchuk et al., 2005). Poleward-directed movement of chromosomes on the metaphase plate and during anaphase is driven by the depolymerization of microtubules attached to their kinetochores (Koshland et al., 1988); (Grishchuk and McIntosh, 2006). Indeed, purified budding yeast kinetochores attached to a single microtubule in an end-on manner can withstand load-bearing forces reaching up to 11 pN (Akiyoshi et al., 2010). When a microtubule, attached end-on to a kinetochore, depolymerizes, it generates a pulling force on that kinetochore and its associated chromosome (Figure 2). The kinetochore proteins can oppose this force if there is sufficient resistance to chromosome movement. In the case where the kinetochores of sister chromatids are attached to microtubules from opposite poles, this resistance is mediated by the cohesin and condensin protein complexes linking the chromatids and the elasticity of the pericentromeric chromatin. In this bipolar configuration, the pulling forces generated at one or both microtubule-kinetochore attachments result in a tension force across the coupled sister kinetochores. This force is analogous to the tension transmitted through a rope pulled from opposite ends. When evaluating potential mechanisms involved in sensing and responding to this tension status, it is relevant to consider that the force is transmitted across the entire linkage, including kinetochore components, centromeric DNA/proteins, pericentromeric chromatin, as well as condensin and cohesin complexes. Thus, any component in this linkage could be involved in sensing and responding to the general tension status of the sister kinetochores.

The role of tension in accurate chromosome segregation has been a fundamental question since the pioneering experiments in the 1960s with the micromanipulation of chromosomes during meiotic divisions in grasshopper spermatocytes (Nicklas and Koch, 1969). Evidence for tension serving as a prominent force within the spindle comes from many studies. For example, analysis of kinetochores on oscillating metaphase chromosomes revealed sites of active and passive force generation (Dumont and Desai, 2012). In fission yeast, severing the microtubules on one side of the metaphase spindle causes the sister chromatids to move toward the spindle pole on the intact side (Klemm et al., 2018). Overall, evidence demonstrates that microtubule depolymerization generates a pulling force on the attached kinetochore, which, in the case of sister chromatids attached to opposite spindle poles, results in a tension force transmitted across the sister kinetochore linkage.

## How microtubule-dependent forces and kinetochore structure lead to chromosome movement

Forces generated by depolymerizing microtubules attached to the kinetochore are vital for creating the pulling forces responsible for chromosome movement during anaphase (Koshland et al., 1988); (Grishchuk and McIntosh, 2006). In addition to depolymerization at the microtubule ‘plus-end’, which is attached to the kinetochore, in most organisms the kinetochore microtubules also undergo a process called flux. This flux is driven by simultaneous polymerization of kinetochore-associated plus-end and depolymerization of the ‘minus-end’ at the spindle pole (Mitchison, 1989). If the depolymerization rate at the pole exceeds polymerization at the kinetochore, it will result in a pulling force toward that pole (Vukušić et al., 2019). The relative contribution of plus-end depolymerization or flux to the overall force experienced at kinetochores varies by organism (Maddox et al., 2000); (Mallavarapu et al., 1999); (Maddox et al., 2002); (Ganem et al., 2005). Overall, microtubule depolymerization at the kinetochore and the poles works to shorten the distance between the spindle pole and the attached kinetochore, thus generating the main forces responsible for poleward chromosome movements and for increasing the tension across bipolar attached sister kinetochores (Inoue, 1953); (Inoué and Ritter Jr, 1975); (Salmon et al., 1976); (Asbury, 2017).

The forces that move chromosomes are significantly larger than the thermally driven background forces in cells and are estimated at 4–5 pN in budding yeast and upwards of hundreds of piconewtons in *Drosophila* cells (Chacón et al., 2014); (Ye et al., 2016). The microtubule tip must be connected perpendicularly to proteins on the outer face of the kinetochore, forming an end-on attachment to withstand such high forces (Asbury, 2017); (Gudimchuk et al., 2020). The KMN network is a group of kinetochore proteins that is essential for forming end-on attachments, and when impaired, results in chromosome segregation defects (Cheeseman et al., 2006); (DeLuca et al., 2005); (DeLuca et al., 2006); (Kim and Yu, 2015); (McCleland et al., 2003) (for review of KMN network see Varma and Salmon (2012). The conserved protein Ndc80, one of the outermost in the KMN network, is needed for microtubule-dependent force production at kinetochores and specifically needed for generating end-on attachments (Wimbish et al., 2020); (Huis in ’t Veld et al., 2019); (Cheeseman and Desai, 2008); (Alushin et al., 2010); (Cheeseman et al., 2006); (Tooley and Stukenberg, 2011); (Suzuki et al., 2016). Much study has focused on the role of the unstructured tail region of Ndc80 in forming attachments, although results indicate this may vary among organisms. While the tail appears dispensable for generating end-on attachments in *S. cerevisiae* and *C. elegans* (Demirel et al., 2012); (Cheerambathur et al., 2013), recent studies using human Ndc80 produced conflicting results (Wimbish et al., 2020); (Huis in ’t Veld et al., 2019). While the exact role of the Ndc80 tail in forming end-on attachment in some organisms remains in question, Ndc80 itself is essential for proper load bearing at kinetochore-microtubule attachments across eukaryotes.

## Microtubule associated proteins strengthen load-bearing capacity of kinetochore-microtubule attachments

In addition to central kinetochore components like Ndc80, several less-conserved MAPs are also essential or aid in forming force-generating microtubule attachments at kinetochores. Many of these are recruited to kinetochores *via* Ndc80 and are important for regulating the attachment to dynamic microtubules (Amin et al., 2019). Highlighted below are MAPs or protein complexes that have been well characterized in their roles to support robust kinetochore-microtubule attachment. For a comprehensive review of MAPs involved in the metaphase spindle and kinetochore-microtubule attachment, see Amin et al. (2019).

In budding yeast, the Dam1/DASH complex is essential for microtubule-kinetochore attachments and dependent on Ndc80 for its kinetochore localization. Although Dam1 appears to be the main microtubule-binding protein in the complex, the nine other DASH subunits are also essential (Westermann et al., 2006); (Asbury et al., 2006); (Grishchuk et al., 2008); (Janke et al., 2001). Mutations in any of the DASH subunits lead to weakened microtubule-kinetochore attachments (Cheeseman et al., 2001). The heterodecameric Dam1/DASH complex oligomerizes to form a ring around the microtubule that is required for persistent kinetochore attachment to dynamic microtubules (Lampert et al., 2010); (Westermann et al., 2005); (Asbury et al., 2006); (Westermann et al., 2006); (Tien et al., 2010); (Tanaka et al., 2007); (Grishchuk et al., 2008). The ring is proposed to function as a collar that can harness the forces produced by depolymerizing microtubules (Figure 2) (Lampert et al., 2010); (Westermann et al., 2005); (Asbury et al., 2006); (Westermann et al., 2006); (Tien et al., 2010); (Tanaka et al., 2007); (Grishchuk et al., 2008); (Kiermaier et al., 2009); (Lacefield et al., 2009); (Lampert et al., 2013); (Miranda et al., 2005); (Umbreit et al., 2014). The rigid, collar-like structure of the ring maintains contact with the microtubule lattice and Ndc80 *via* flexible C-terminal extensions of the Dam1/DASH complex (Jenni and Harrison, 2018). This flexible connection could potentially accommodate different kinetochore-microtubule configurations, such as lateral *versus* end-on attachments during the cell cycle. In addition to mediating forces, the Dam1/DASH complex also regulates microtubule-kinetochore attachments *via* its phosphorylation status. Phosphorylation of the DASH subunit Ask1, by Cdk1, promotes robust microtubule-kinetochore attachment, likely by promoting Dam1/DASH complex oligomerization (Gutierrez et al., 2020). When microtubule-kinetochore attachments are not under sufficient tension, the budding yeast Aurora B homolog, Ipl1, phosphorylates Dam1/DASH complex components to weaken interactions with the microtubule and promote detachment (discussed below) (Keating et al., 2009); (Cheeseman et al., 2002).

Two budding yeast MAPs conserved across eukaryotes also function at force-generating microtubule-kinetochore attachments. The first MAP is Stu2, a well-characterized member of the XMAP215 family that has orthologs in many organisms, with human (chTOG), fission yeast (Dis1), worm (Zyg9), and frog (XMAP215) being among the best described (Amin et al., 2019). XMAP215 family members play prominent roles in controlling the dynamic behavior of microtubules in many cellular processes, including at kinetochores (Amin et al., 2019). Work in budding yeast has shown that Stu2 localizes to kinetochores, where it interacts with Ndc80 (Miller et al., 2016). Disrupted Stu2 kinetochore localization results in microtubule attachment defects, with data indicating Stu2 functions in the establishment of bipolar attachments and stabilizes kinetochore-microtubule attachments under tension (Miller et al., 2019). The second MAP is the homotetrameric kinesin-5 motor protein, Cin8. Kinesin-5 motors are well known for their role in crosslinking and sliding interpolar microtubules in the spindle. Cin8 also localizes to the region of kinetochore-microtubule attachment in an Ndc80-dependent manner (Suzuki et al., 2018). In the absence of Cin8, kinetochores experience less tension as measured by a tension-sensitive FRET module placed within Ndc80. Additional data suggest that Cin8 may promote tension by delivering Protein Phosphatase 1 (PP1) to kinetochores, where it dephosphorylates Ndc80, thus increasing the strength of microtubule attachments (Suzuki et al., 2018).

In metazoan cells, the Ska complex (SKA1, SKA2, and SKA3/Rama1) significantly strengthens microtubule-kinetochore attachments (Auckland et al., 2017); (Gaitanos et al., 2009); (Theis et al., 2009); (Welburn et al., 2009); (Ohta et al., 2011). The Ska complex is proposed to be a functional ortholog of the Dam1/DASH complex in fungi (Welburn et al., 2009); (Van Hooff et al., 2017). When Ska complex localization to kinetochores is prevented *in vivo via* Ska3 depletion, cells experience a large increase in mitotic duration, indicating difficulty in establishing robust, tension-generating attachments (Zhang et al., 2017). *In vitro* work has shown that the Ska complex increases the load-bearing capacity of Ndc80-based attachments by binding to both Ndc80 and the microtubule. Using Ndc80 with mutations in the tail region, which lowers affinity for the microtubule, the Ska complex was able to enhance the attachment strength by as much as five-fold (Helgeson et al., 2018); (Huis in ’t Veld et al., 2019). The Ska complex can produce robust attachment, independent of Ndc80 tail phosphorylation status, suggesting it can compensate for the tail-mediated regulation of microtubule binding and perhaps antagonize Aurora B, which phosphorylates the Ndc80 tail to promote the release of tensionless kinetochore attachments (discussed below) (Helgeson et al., 2018); (Wimbish et al., 2020).

Another factor implicated in kinetochore-microtubule attachments in human cells is the Astrin-SKAP complex. Knockdown of Astrin results in disrupted spindle organization and mitotic delay, indicating a vital role(s) in mitotic spindle formation (Gruber et al., 2002). The Astrin-SKAP complex binds microtubules throughout the cell cycle (Kern et al., 2016) yet only localizes to kinetochores once they achieve bipolar attachments in late metaphase (Fang et al., 2009); (Schmidt et al., 2010); (Friese et al., 2016); (Mack and Compton, 2001); (Manning et al., 2010). Astrin-SKAP localization is important for chromosome alignment and maintenance of sister chromatid cohesion (Thein et al., 2007); (Manning et al., 2010); (Dunsch et al., 2011). The localization and function of Astrin-SKAP are inversely related to Aurora B activity, suggesting Astrin-SKAP may antagonize the attachment-destabilizing activity of Aurora B (Fang et al., 2009); (Schmidt et al., 2010). Along those lines, Astrin-SKAP has been shown to facilitate the conversion of kinetochores associated with the lateral side of a microtubule to the end-on configuration (Shrestha et al., 2017). However, the conserved tail of Astrin has been shown to direct PP1 to kinetochores, where PP1 stabilizes microtubule-kinetochore attachments *via* a mechanism that appears independent of Aurora B activity (Conti et al., 2019). Astrin also interacts with Polo-like Kinase 1 (Plk1), whose phosphorylation of Astrin promotes its kinetochore localization and attachment stabilizing activity (Geraghty et al., 2021). Imaging of mitotic cells indicates that the microtubule attachments of sister kinetochore pairs are under higher tension in cells lacking SKAP (Rosas-Salvans et al., 2022). Moreover, kinetochores move slower on polymerizing and depolymerizing microtubules, and more force is needed to convert shortening kinetochore-attached microtubules back to growth. These observations suggest that Astrin-SKAP works to preserve bipolar attachments by reducing friction or effectively ‘lubricating’ their kinetochore-microtubule connections (Rosas-Salvans et al., 2022). Altogether these findings demonstrate that there are likely multiple layers of tension-regulating mechanisms at the kinetochore, which help to produce more robust attachments in combination.

## The roles of tension in promoting accurate chromosome segregation

**FIGURE 3:**
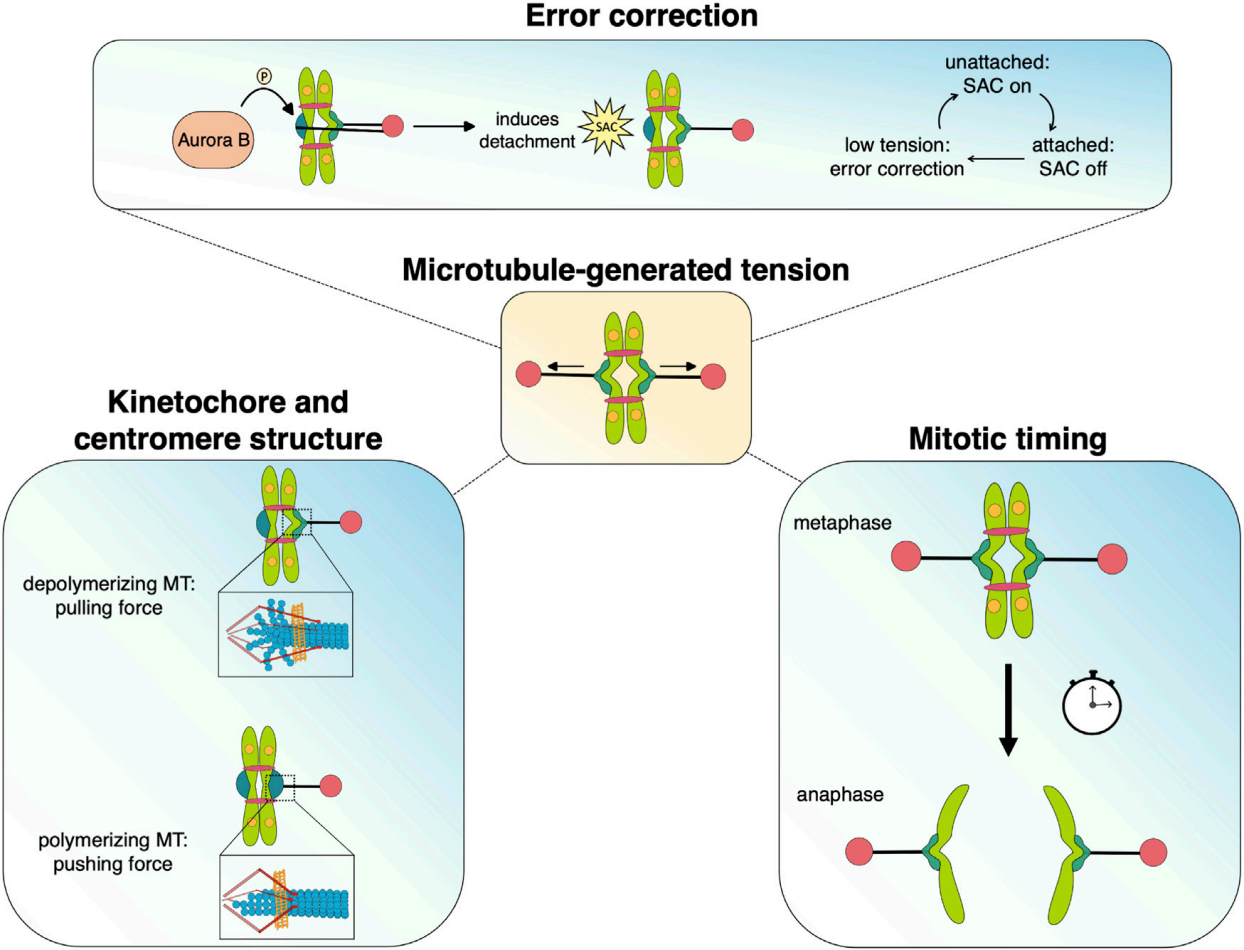
Microtubule-generated tension serves as a unifying force that facilitates the processes that promote chromosome segregation during mitosis. Dynamic microtubules generate pushing and pulling forces that move chromosomes as well as alter kinetochore/centromere structure, which may stabilize attachments and silence spindle assembly checkpoint signaling. The tension status at kinetochores also mediates Aurora B-dependent error correction and regulates the timing of anaphase onset.

When unable to generate robust tension across sister kinetochore attachments, cells face prolonged mitotic duration and increased risk of chromosome missegregation, leading to aneuploidy or death. Dynamic microtubules generate robust tension with end-on attachments, whose coupling and regulation requires the function of specialized kinetochore components and MAPs, prominent examples of which are described above. Below we summarize recent advances in understanding how tension can act as a unifying factor, connecting events from the pericentromeric region to the outer kinetochore. Tension can alter kinetochore and centromere structure, regulate attachment strength, act as a modulator of Aurora B activity, and, in conjunction with the Spindle Assembly Checkpoint (SAC) promote timely anaphase onset (Figure 3). Tension across sister kinetochores is a fundamental quality of bipolar attachment that plays a key role in multiple mechanisms and thus unifies their individual functions to achieve accurate chromosome segregation.

### Tension directly influences kinetochore and centromere structure

The tension produced across sister kinetochores during metaphase is a physiologically relevant force generated within the mitotic spindle (Chacón et al., 2014); (Ye et al., 2016). It has been a long-standing endeavour to elucidate how this tension status is sensed at kinetochores and centromeres. Notably, the structures of both kinetochores and centromeres are influenced by tension. Centromeres are the singular regions of DNA where kinetochore proteins assemble and they serve as a key connection point between replicated sister chromatids (for a review of centromere structure, see Lawrimore and Bloom (2022). It has long been thought that cohesion between sister chromatid DNA strands, provided by the cohesin complex, functions as the major mechanism for resistance to outward forces on their centromeres. One apparent limitation is that cohesin complexes can move relative to the associated DNA and, thus, allow centromeres to be pulled apart with relatively little resistance due to such DNA sliding. This challenge is met by ‘trapping’ the cohesin complexes between a pair of convergently oriented genes on either side of the centromere, thus limiting further DNA sliding and defining the boundaries of the pericentromeric region (Paldi et al., 2020). A notable property of DNA is that it is relatively floppy and easily extended, which does not generate much tension until it is largely extended (Bloom, 2008). Although alternative models have been proposed for the physical arrangement of the pericentromeric DNA between sister centromeres (Paldi et al., 2020), considering the length of DNA within the pericentromere, the bottlebrush model perhaps best accounts for this extensible property of DNA (Lawrimore et al., 2015). Briefly, the bottlebrush model posits that the pericentromeric DNA is organized by condensin and cohesin complexes into a looped loop structure with a central backbone and extending loops resembling the bottlebrushes used to clean test tubes (reviewed in Lawrimore and Bloom (2022). Notably, the bottlebrush model provides a mechanism by which the pericentromeric DNA adopts a stiff structure that allows for microtubule-dependent kinetochore movements to result in relatively greater tension. Centromeric stiffness increases during mitotic progression, with the greatest stiffness during metaphase (Harasymiw et al., 2019). This increased centromeric stiffness in metaphase, when tension is a vital signal for achieving bipolar attachments, passively increases tension in response to active microtubule-depolymerization forces, relative to centromeres with more extendable DNA. This centromeric stiffness creates a more sensitive mechanism to distinguish different kinetochore-microtubule configurations and ensure bipolar attachments are formed (Harasymiw et al., 2019). Altogether, the centromere and associated proteins are critical for proper tension generation and for enhancing the tension-responsive signaling that ensures bipolar kinetochore-microtubule attachments are established.

The kinetochore itself is a mechanically rigid structure compared to DNA. It has been proposed that specific proteins, such as Ndc80, are physically extended by microtubule pulling forces and thus provide a potential mechanism to report the kinetochore tension status (Suzuki et al., 2016). A related idea is that multiple kinetochore proteins or complexes change conformation or position relative to others in response to pulling forces, again serving as a physical indicator of tension (Joglekar et al., 2009); (Maresca and Salmon, 2009); (Uchida et al., 2009); (Wan et al., 2009). Indeed, a tension-dependent change in the shape of inner kinetochore proteins, particularly CENP-T, which undergoes elongation, has been observed in chicken DT40 cells (Suzuki et al., 2011). A FRET-based study using fluorescently labelled Ndc80 and kinetochore microtubules in human U2OS cells found that the number of Ndc80 molecules bound to kinetochore microtubules increased with tension (Yoo et al., 2018). A similar approach demonstrated that the Ndc80 clustering to individual microtubules is comparable at human and yeast kinetochores (Kukreja et al., 2020). Moreover, a distinct structural response has been observed between kinetochores that have lost tension *versus* those that lost attachment (Roscioli et al., 2020). KNL1 is shown to unravel at the loss of tension, while NDC80 jackknives due to microtubule detachment (Roscioli et al., 2020). This is unique to the other outer kinetochore proteins, which have high nematic order and do not undergo significant structural change in response to loss of tension or attachment (Roscioli et al., 2020). Altogether this evidence supports the idea that physical changes at the kinetochore serve as mechanical cues for tension-dependent processes in mitosis.

### Tension regulates Aurora B and strengthens bipolar attachments

Aurora B (Ipl1) is needed to respond to tensionless kinetochores and is the major kinase involved in the associated error correction process (Chan and Botstein, 1993); (Biggins et al., 1999); (Cheeseman et al., 2002); (Tanaka et al., 2002). Error correction is the mechanism by which kinetochore-microtubule attachments experiencing insufficient tension are selectively destabilized, thus granting another chance to establish force-generating bipolar connections. It is mediated by Aurora B *via* phosphorylation of KMN proteins, such as the tail of Ndc80 (Biggins et al., 1999); (DeLuca et al., 2006); (DeLuca et al., 2011); (Welburn et al., 2010), as well as components of the Dam1/DASH complex (Tien et al., 2010); (Cheeseman et al., 2002). While Aurora B phosphorylates other spindle proteins, such as MAPs associated with the spindle midzone, the modification of kinetochore proteins facilitates error correction. Phosphorylation of the kinetochore components decreases their affinity for the microtubule, weakening the kinetochore-microtubule linkage and promoting detachment (Welburn et al., 2010); (Sarangapani et al., 2013). Conversely, the presence of sufficient tension avoids triggering error correction and, thus, selectively stabilizes bipolar microtubule-kinetochore attachments (McVey et al., 2021). In addition to avoiding Aurora B-mediated error correction, the kinetochore-microtubule linkage behaves like a catch bond in that its binding strength is enhanced under pulling forces (Akiyoshi et al., 2010). As a result, tension-generating bipolar attachments are inherently stabilized.

The mechanism by which Aurora B specifically targets tensionless kinetochore-microtubule attachments, yet avoids those under tension, remains elusive. There are several proposed models for Aurora B activity in response to insufficient tension at kinetochore attachments (for a review of models of Aurora B activity, see McVey et al. (2021). Two well-established classes of models are the spatial dependent (Liu et al., 2009) and the tension-sensitive activation models (Sandall et al., 2006); (Adams et al., 2000); (Bishop et al., 2005). In the spatial dependent models, once Aurora B is localized to the inner centromere, it is constitutively active and will phosphorylate any kinetochore substrate that comes within range. Thus, the components of sister kinetochores that lack bipolar pulling forces and the resultant tension to be sufficiently displaced away from the centralized Aurora B, will be modified, triggering detachment. Conversely, if microtubule-generated forces can pull sister kinetochores far enough apart, concomitantly producing tension, their components will be physically displaced out of the zone of Aurora B phosphorylation, which indirectly stabilizes the attachments. In the activation models, Aurora B activity is modulated by the tension status rather than the proximity of kinetochore substrates. Although Aurora B may encounter its kinetochore substrates, its kinase activity would be inhibited by higher tension or stimulated by low tension. In budding yeast, tension sensing and error correction by Aurora B can occur in the absence of its centromere localization, supporting the hypothesis that Aurora B activation is triggered by tension status (Campbell and Desai, 2013). Further work consistent with the activation model using purified yeast kinetochores suggests that tension-generating attachments can directly regulate Aurora B activity or oppose its outcome (de Regt et al., 2022). Another emerging model, supported by recent findings, is that Aurora B localization to the kinetochore itself, as opposed to the inner centromere, functions to phosphorylate substrates on low-tension kinetochores, while the kinase is evicted from kinetochores that establish tension (Reviewed in (Broad and DeLuca, 2020). This mechanism bears similarity to the activation model in some respects, particularly if Aurora B would be recruited back to kinetochores that subsequently lose tension. In addition to canonical models where Aurora B activity mainly promotes detachment, a recent study provides evidence that the tension status influences the downstream effect of Aurora B phosphorylation. Namely, under low tension Aurora B caused kinetochore microtubules to depolymerize without detachment, but under high tension, the microtubules detach, a difference which the authors propose may be relevant to correcting distinct attachment errors (Chen et al., 2021).

### Tension and the spindle assembly checkpoint work together to promote timely metaphase-anaphase transition

Much work has been done to understand how microtubule-generated tension at kinetochores and the SAC work together or independently to promote accurate chromosome segregation. These questions have been difficult to approach experimentally due to the challenge of isolating the effect(s) of the tension status from kinetochore attachment. Very briefly, conditions that reduce tension can also inhibit attachment or induce error correction-mediated detachment, while those preventing attachment also preclude tension. In the presence of unattached kinetochore(s), the SAC will delay anaphase onset (for a full review of the SAC, see (Lara-Gonzalez et al., 2021). Phosphorylation of the kinetochore protein Spc105/KNL1 at unattached kinetochores by Mps1 promotes localization of the Bub (Bub1, Bub3) and Mad (Mad1, Mad2, and BubR1/Mad3) proteins (London et al., 2012); (London and Biggins, 2014). This kinetochore localization leads to catalytic formation of the Mitotic Checkpoint Complex (MCC), which inhibits the Anaphase Promoting Complex (APC/C) by sequestering Cdc20, a required activator of the APC (Lara-Gonzalez et al., 2021).

SAC signalling is necessary to provide sufficient time to establish proper kinetochore-microtubule attachments yet also allows for timely mitotic progression. Along these lines, experiments in budding yeast suggest Bub1 works with the well-established tension-sensitive protein, Sgo1, and the phosphatase PP2A to prevent premature SAC silencing (Jin et al., 2017). On the other hand, recent data indicates that Bub1 and Aurora B work cooperatively to maintain SAC signalling once initiated, even after Mps1 activity at kinetochores has diminished (Roy et al., 2022).

One outstanding mystery, owing to the fact that unattached kinetochores are inherently tensionless, is whether the tension status plays any role in the canonical SAC mechanism at unattached kinetochores. It has been postulated that tension plays a key role in signalling the establishment of proper attachments, and thus silencing the SAC. Although significant work has been directed at understanding how SAC signalling is extinguished once appropriate conditions are met, the mechanistic basis remains largely obscure.

An important step in SAC activation is Mps1-mediated phosphorylation, and thus, silencing the SAC could entail preventing Mps1 phosphorylation of Spc105/KNL1. One potential mechanism is that microtubules and Mps1 competitively bind Ndc80, with microtubule binding displacing Mps1, thus silencing the SAC (Hiruma et al., 2015); (Ji et al., 2015). This competitive binding model has been challenged by work done in *Drosophila* demonstrating that as microtubule attachments are established, Mps1 localization at kinetochores decreases, except for a small fraction that remains on kinetochores until anaphase onset (Moura et al., 2017). Follow up work revealed that Mps1 localization to syntelic, end-on attached kinetochores proceeded their detachment (Hayward et al., 2022). Mps1 transiently localizes to these kinetochores, which have high levels of Aurora B phosphorylation, to promote timely error correction (Hayward et al., 2022).

Another potential mechanism is that the SAC is silenced by tension-dependent kinetochore stretching, and the sustained deformation of kinetochore components at the attachment interface (Maresca and Salmon, 2009); (Uchida et al., 2009). Other work in budding yeast has posited a mechanical switch in the kinetochore upon end-on attachment that prevents SAC signaling from persisting, where by Mps1 is prevented from phosphorylating Spc105 (Aravamudhan et al., 2015). In this model, Dam1 acts as a barrier preventing Mps1 access to Spc105 (Aravamudhan et al., 2015).

In general, it is thought that extinguishing SAC signaling, regardless of mechanism, entails either eviction of Mps1 from the kinetochore or a decrease in Mps1 activity. In *Drosophila*, Mps1 activation is regulated by PP1-87B phosphatase activity that antagonizes the increased Mps1 activity resulting from T-loop autophosphorylation (Moura et al., 2017). In human cells, this T-loop autophosphorylation, as well as phosphorylation by Aurora B that impacts Mps1 kinetochore localization and activation, is antagonized by PP2A-B56 (Hayward et al., 2019). In both cases, a decrease in phosphatase activity leading to persistent Mps1 T-loop phosphorylation and Mps1 activity resulted in prolonged mitotic arrest (Moura et al., 2017); (Hayward et al., 2019). Altogether, phosphoregulation of Mps1, resulting in its activation or kinetochore localization, is mediated *via* PP1-87B and PP2A-B56 to promote SAC silencing and timely anaphase onset.

Recent work has generated conflicting evidence for whether tension is needed to silence the SAC or if microtubule attachment alone is sufficient. PP1 is a phosphatase that antagonizes Mps1 activity at the kinetochore *via* dephosphorylation of kinetochore substrates. In HeLa cells, intra-kinetochore stretching is diminished in monopolar spindles, leading to decreased PP1 recruitment to kinetochores (Uchida et al., 2021). At these PP1 deficient kinetochores, downstream SAC proteins remained, leading to delayed anaphase onset, irrespective of Mps1 localization (Uchida et al., 2021). This work suggests that tension is needed for kinetochore stretching to silence the SAC. On the other hand, evidence indicates that a lack of tension can, in certain cases, activate the SAC. In HAP1 cells with deficient Kif18A, a kinesin-8 motor, the resulting lack of tension activates the SAC (Janssen et al., 2018). At Mad1 positive kinetochores in these cells, tubulin/microtubule signal was equivalent to that in control cells, demonstrating that kinetochores were fully attached even though the SAC was active (Janssen et al., 2018). These findings indicate that insufficient tension can activate the SAC, regardless of attachment status (Janssen et al., 2018). In budding yeast, deletion of the conserved kinesin-5, Cin8, results in a delay in anaphase onset due to a lack of tension *via* Ndc80 (Suzuki et al., 2018). This reduced tension is associated with sustained error correction-mediated phosphorylation of Ndc80 due to disrupted kinetochore recruitment of PP1 to antagonize Ipl1 activity, resulting in detachment and SAC activation (Suzuki et al., 2018). Together these studies reveal that the tension generated by end-on attachments is a signal that potentially facilitates multiple mechanisms that activate or silence the SAC.

Contrary to tension being a central signal, other evidence suggests that attachment is the major signal needed to satisfy or silence the SAC. Cells expressing a non-phosphorylatable Ndc80 tail mutant (Hec1-9A) can establish stable end-on attachments. Notably, when these cells are induced to form monopolar spindles, they are unable to generate the normal tension associated with bipolar attachments yet still establish stable end-on connections (Etemad et al., 2015). Although the SAC appears functional in these cells, the low-tension attachments can sufficiently satisfy the checkpoint (Etemad et al., 2015). In another study, Hec1-9A and wild type cells were treated with low dose nocodazole to deplete poleward pulling forces (Tauchman et al., 2015). While nocodazole treatment arrested wild type cells, the Hec1-9A cells satisfied the SAC and entered anaphase, which was corroborated by a decrease in Mad1 positive kinetochores (Tauchman et al., 2015). Additional evidence in human cells show that low kinetochore-microtubule occupancy, or fewer microtubules bound to a kinetochore compared to the typical number at those “fully attached”, does not impact SAC silencing (Etemad et al., 2019). SAC proteins were almost undetectable at kinetochores, despite only about half the total possible microtubules bound to kinetochores (Etemad et al., 2019). This body of work suggests that microtubule-kinetochore attachment is sufficient to silence the SAC, regardless of maximum kinetochore occupancy or tension-generating status. These experiments represent significant technical advances that continue to take us closer to understanding how tension contributes to signalling at kinetochores. They also highlight the intricate relationship between attachment and tension at kinetochores, and the challenges in elucidating the role of each in SAC activation or silencing.

While it remains unclear how or if tension contributes to SAC signalling, the tension status can impact anaphase onset independently of SAC silencing. Work in budding yeast shows Bub1 and Bub3 function outside of their canonical SAC role to delay anaphase onset in response to low-tension but attached kinetochores (Proudfoot et al., 2019). Thus, a tension-sensitive mechanism may provide extra time for kinetochores to come under tension prior to anaphase or to trigger error correction to sample for a bipolar attachment (Proudfoot et al., 2019). Altering the level of tension generated in the spindle by deleting specific MAPs (Cin8, Kip1, Ase1) has been shown to produce a graded response, where detachment mediated by kinetochore protein phosphorylation *via* Aurora B is enhanced by decreasing centromeric tension (Mukherjee et al., 2019). This work reveals that tension-sensitive signalling mechanisms can be sensitive to the magnitude of forces experienced.

While phosphorylation has been a widely investigated post-translational modification that mediates signalling at centromeres and kinetochores, SUMOylation of proteins has recently been found to also play a role. The SUMOylation status of Sgo1, a well-established tension-sensitive protein, influences the timing of anaphase onset (Su et al., 2021). Sgo1 is recruited to the pericentromeric region during metaphase by phosphorylation of S121 on histone H2A (Fernius and Hardwick, 2007); (Yamagishi et al., 2010). This positioning allows Sgo1 to promote chromosome biorientation by facilitating Chromosome Passenger Complex (CPC) localization to the centromere and recruiting cohesin and condensin (Nerusheva et al., 2014); (Verzijlbergen et al., 2014); (Peplowska et al., 2014). Sgo1 SUMOylation is needed to keep sister chromatids in a stable bioriented state. Metaphase arrested cells harbouring a mutation in the coiled-coil region of Sgo1 (sgo1-4R) that reduces SUMOylation display cycles of biorientation, loss of tension, detachment, then reattachment to become bioriented again (Su et al., 2021). Even with multiple rounds of unnecessary error correction, these cells have significantly lower chromosome missegregation levels than those lacking Sgo1. Together the results suggest that SUMOylation of Sgo1 and the CPC component Bir1 work to decrease Sgo1 localization, and thus dampen Aurora B (Ipl1)-mediated error correction to selectively stabilize bioriented kinetochore-microtubule attachments and promote timely anaphase onset (Su et al., 2021). More work is needed to understand how the tension status is transmitted to these downstream effectors.

## Concluding remarks

While multiple classes of microtubule- and motor-dependent forces contribute to formation of the bipolar metaphase spindle, microtubule depolymerizing forces at end-on kinetochore attachments are vital for ensuring accurate chromosome segregation. The tension generated across bipolar attachments serves as a unifying factor linking events from the centromere to the outer kinetochore, including 1) modulating centromere and kinetochore structure, 2) mediating Aurora B activity, and 3) working with the SAC to regulate the timing of anaphase onset. While we have made significant advances in understanding the role of tension in chromosome segregation, there are many questions that remain to be answered. For example, what is the mechanism by which tension status is converted to cellular signalling? How does the tension status initiate, sustain, and/or silence the SAC? To what extent are the mechanisms that sense attachment and tension shared or independent? Despite over 50 years since the importance of tension in chromosome segregation first captivated biologists, elucidating its role(s), both mechanistically and at the molecular level remains challenging. Further advances in genetic and experimental approaches will likely be needed to clearly discriminate the roles of tension and attachment at the kinetochore.
